# On the Pathogenetic Effects of Malaria

**Published:** 1864-03

**Authors:** W. G. Munson


### ON THE PATHOGENETIC EFFECTS OF MALARIA.

By W. C. MUNSON, M. D.

Twenty years experience in the practice of medicine, in the Mississippi Valley, has convinced the writer that all diseases induced by malaria, or miasma, have, in common, *one* distinguishing characteristic, viz: an abnormal reduction, or depression, of *vital force* or *power*.

In the following remarks the terms malaria, miasmas and mephitic-poison are used synonymously. "The term miasma has been very properly used to signify exhalation." There are certain exhalations from marshes, which have been supposed to cause several varieties of fever, viz: Intermittent, Remittent, Pernicious, etc.; hence the name, "Malarial fevers."

It has never been clear to my mind that all the diseases which have been ascribed to malarial causes have been thus produced. And the question is suggested, may not *other* causes, such as exposure to cold, sudden transitions from cold to heat, damp atmosphere, etc., produce disease which in its symptoms so nearly resemble that caused by *malaria* as to be readily confounded with it? May not our natural proneness to jump at conclusions without sufficient evidence have led us into frequent error on this subject? Because many cases are found to be clearly of miasmatic origin, it has perhaps been taken for granted that all this class of diseases have been produced by the same cause. And the error may the more readily be excused, when we take into consideration the fact that a vast majority of febrile diseases in this country can be clearly traced to mephitic poison. Indeed the clinical experience of most practitioners in the Mississippi Valley has convinced them that a vast majority of the diseases they meet with do thus originate. And doubtless the above pathological error has been the cause of much *seeming* contradiction in the clinical experience of so many learned members of the profession as regards therapeutics. And it is believed that a little more attention to the pathology of these diseases will enable us to distinguish pretty clearly diseases of malarial origin from all others, and thus be able to know the reason why in a few cases—and only a few—of febrile diseases, of the present day, the *antiphlogistic* treatment is imperatively demanded; while, in so many more, it is contra-indicated.

It is readily admitted that this can only be done by noticing carefully the effects of *malarial poison* on the system. All attempts to analyze and classify the peculiar poisonous elements which produce these diseased conditions will, perhaps, fail, as they have done heretofore. They have been supposed to originate from *cryptogamia* of animal or vegetable germs. But this hypothesis appears to lack confirmation. It is true that the concurrence of heat moisture and vegetable decomposition appears necessary to generate miasmatic poison.

The summer of 1854 was a very hot and dry one; so much so that many ponds and marshes were completely dried, exposing surfaces of soil favorable to cryptogamous growths; this was followed by the summer of 1855, so noted as a sickly season throughout the country, and which was hot and wet, favorable to the development and maturity of the above growths. During the rains of the latter part of this summer, and early part of the fall, almost invariably after each rain occurring in the night, a green substance was found covering the ground in the morning. Some of this the writer examined under the microscope, and found it to consist of microscopic vegetation and *sporule.* On placing some of it in cold rain water, which had been previously boiled and filtered, in two days time the water was filled with *monadal infusoria.* Whether such a condition of the atmosphere will produce disease or not, I am not prepared to say. It probably will, however.

Again, on the other hand, the old notion that carbonic acid gas is the poisonous agent, has never been abandoned. And observations in marshy districts, with the present aids of science, seem to corroborate this view. It has been observed for many years that sickly seasons are usually preceded by copious and long-continued spring rains. The marshes, ponds and low grounds are thus filled with water; under water the vegetation undergoes partial decomposition; the *carbon*, meantime, not readily escaping. Consequently its injurious effects are not much felt until, the rains having ceased, the hot summer's sun evaporates the water and leaves large masses of decayed and decaying vegetation exposed. The exceedingly unpleasant odor so universally observed to arise from marshes, especially after sundown, is thus explained. It is thought that the sensible increase of stench at this time is attributable to the carbon of the partially decomposed vegetation, set free by the rapid evaporation consequent on a hot summer day. It is assisted by the heat of the sun during the day to commingle with the atmosphere as it is set free. But when the heat is withdrawn it remains in excess for a time, until by the law of the commingling of gases it is taken up. It is argued that the peculiar sickening *odor* and sense of oppression felt on inhaling this poison under these circumstances, may be caused by this gas, modified, perhaps, by the circumstances which produce it in excess. Again, it has been observed for many years on our western prairies, that large tracts of recently "broke up prairie" appear to generate malaria. Families have been known to live for years on the "open prairie" with not a case of fever appearing among them during the time; but, an enterprising neighbor comes in and turns up the "sod" of several hundred acres in the immediate vicinity, and now, in the neighborhood of this large "breaking," they are all "down with the chills." And on passing this spot in the evening, after a hot day, much the same disagreeable odor and oppressive sensation is experienced as on passing a marsh in the evening. It is believed that the poison so tangibly felt under these circumstances is a peculiar quality of carbonic gas, set free from decomposing vegetable matter, in excess.

The objection, that *carbon*, when inhaled, does not produce the effects known to follow the inhalation of miasmatic poison, is claimed to be of no force, because it has not been and perhaps cannot be shown, that the gas in question, somewhat modified, or perhaps by some catalysis, is not capable of producing all these effects. It appears to be pretty well established that the same exhalations—modified, perhaps, only by the degree of their intensity, or the idiosyncracy of the subject—produce Intermittent, Remittent, Pernicious, and other fevers.

On close attention to this subject, it will be found that there is at least *one* remarkable coincidence between the effects of malaria on the system and *carbon*, to wit: a *sensible* feeling of depression. A result, it is thought, of a reduction of the vital *force* or *action*.

It may not be of any great practical importance, however, to determine whether the poison in question is gas, infusoria, or something else; but careful clinical observation on the *effects* of the poison, must be regarded as essential.

"Life cannot be considered an abstract entity." The term *forces*, or powers of life, are understood to be used metaphorically. Nevertheless, there is what, for the sake of convenience, we will call *vital action;* which may be explained to be the effect of an unknown agency by which life is sustained, and from which results voluntary and involuntary motion, sensation and reproduction; in short, that which sustains animal life and prevents chemical decomposition of animal organisms. There appears to be three *distinct actions* continually operating in animal beings, viz: the *vital*, already explained; the *chemical*, which is continually producing decomposition, or pulling *down*, as well as reconstruction, or building *up*; and, perhaps, what it may be proper to call *electrical action*.

These actions, as is well understood, are severally and conjointly comprehended by the term, "*Chemical vital*" action, or "*Biochymia*." But it is thought by the writer, that they each operate to perform distinct and important functions; that they are continually operating to sustain animal life, and that each is kept in equilibrium by the action of the other, or, at least, that the *chemical* is controlled or kept in due bounds by the *vital*. For it is observed that when the vital ceases to act, destruction of the organism is the consequence, by *chemical action*. Clinical experience has abundantly shown that "malarial poison" has the effect, directly, to depress, or reduce the "vital power or action." How? we may not be able to say. Yet the fact can not be doubted. It may be that, by inhalation, the poison comes in contact with the blood in the lungs, and an organo-chemical change takes place in that vital fluid, so that the material, perhaps, for supplying the waste of organs, is not conveyed to them. The writer inclines to the opinion, however, that the effect is first produced on the cerebro-spinal system; and, by directly reducing the vital power, impedes the entire functions of life. The blood soon becomes impoverished, perhaps, by some chemical action; the liver impaired in its functions, as well as the entire glandular system; and, perhaps, the whole system, even to the ultimate cell structures. The old liver, though slighted, and often too much neglected in these latter days of progress, is still deemed worthy of attention.

Prof. Geo. Hastley, in a paper read before the "Royal Medical and Chirurgical Society," and noticed in the London *Lancet*, says: "The liver is the great laboratory for the manufacture of blood corpuscles in man. Hence the *anœmia* which so speedily follows the suppression or impairment of its functions."

This depressing effect of the poison in question—judging from its effects—impairs the vital action, and, as a consequence, an increased chemical action follows. And as all chemical action, whether in organic or inorganic matter, evolves heat, and more or less electricity, and this electricity, it is believed, comes in aid of the vital power, and prevents serious organo-chemical decomposition. Electrical action is a result of, or is connected with chemical action, in which, during the process of composition and decomposition of the various animal tissues, electricity is developed to be either retained in the system or set free; and which, in many of its actions, bears a close analogy, almost amounting to absolute identity, with vital action; and, perhaps, for which it may act as a substitute sometimes, and thus preserve health and life, where the vital action is impaired. Vital action may be, for all that is now known, closely allied to electricity, possibly dependent on it. It may be hoped that much that is important in pathology, may yet be learned, by investigating the subject of electricity in its connection with animal life. Health consists in an equilibrium, or correct proportion of these several actions in the system, and when there is a derangement of this equilibrium, a lesion of the living tissues results from an increased chemical action, and disease or death ensues. Chemical action appears always to increase in an inverse ratio to the amount of depression, or sinking of the vital. The phenomena of fever, is believed to illustrate this view. The cold stage, or chill, it is assumed, is the direct effect of depression; and now, as the vital power is depressed, the chemical action preponderates. If not enough to produce perceptible organic lesion ; yet always enough to produce functional derangement. And the tissues involved undergo these chemical changes according to the reduction of their vital energy. But this chemical action—as before observed—evolves heat and electricity. And electricity being the best substitute for vital action with which we are acquainted, assists in preserving the system from the more deadly influence of chemical decomposition until its normal functions are restored. It also contributes to augment the temperature of the body, and thus produces—or contributes to—that exacerbation of heat called fever. Perhaps death would result from each chill which precedes fever, was it not for the increased chemical action which, by its accompanying electricity, now comes in aid of the vital powers, and thus prevents farther destruction, until by reaction the vital action is again established.

If the above positions should come to be regarded as established facts, an important step will have been taken in pathological science, and consequently in therapeutics.

This hypothesis, if true, throws much light on certain facts established by clinical experience, but never fully explained. Such as, the unusually rapid dissolution attending the diathesis popularly known as congestive chills. These cases appear to be suddenly fatal, either from the virulence of the poison, or from the inability of the system to resist it. Consequently, the reduction of the vital power is so sudden and complete, that reaction does not take place; and the lesion caused by chemical action uncontrolled by the vital, results in the speedy death of the patient. And again, in inflammatory fevers of the asthenic type, a superabundance of *fibrine* has been found in the blood. And M. Trousseau, in a recent lecture in Paris, pointed out the striking analogy existing between the more serious symptoms of typhoid fever and those consequent on protracted abstinence, that in both, the blood has an excess of fibrine. It is suggested that fibrine may exist in the blood, not only as material for rebuilding and supplying the waste of organs, but it may also exist as the *debris* of wasting, or impaired organs—*i. e.*, cast off material in process of elimination from the system. Its presence so largely in the blood of animals undergoing starvation, seems to corroborate this view. Hence the anæmia so rapidly following in fevers of this type; and the conclusion is thus strengthened, that in all diseases of malarial origin, whether of intermittent, remittent, or typhoid type, the asthenic condition will be manifest, and that the excess of fibrine in the blood in these conditions is referable to the lesion of the living tissues by chemical metamorphosis, resulting from lack of vital action.

Fever, then, should be regarded, not so much as a disease, as an "effort of nature," to throw off disease. And y t, it is manifestly absurd to call it a "healthy action." Because, unquestionably, the condition is abnormal, and therefore should not be called healthy. If the above be the true pathogeny of malarial diseases, the adaptation of therapeutics to this pathogenetic view, must be regarded as essential. And the truth of the terse, and somewhat eccentric remark of a certain writer, is brought to mind, that "the patient should always be treated instead of the disease."

The clinical experience of so large a number of observing practitioners, both in Europe and America, coinciding in the rejection of the antiphlogistic treatment in so many cases of inflammatory and pernicious fevers, may be claimed as corroborative of this hypothesis.

The following remarks, made by M. Herard, in a clinical lecture at the "Hotel Dieu," at Paris, bear upon this point, "The treatment of typhoid fever is, of course, different, according to the theory adopted on the nature of the disease. Those who adopt the theory of follicular inflammation of the intestinal tube—an ulcerous affection of Peyer's glands—as the cause of the disease, consistently apply antiphlogistic remedies."

In the opinion, however, that this inflammation, or the decomposition of local secretions, and consequent absorption of poisonous fluids calculated to induce a septic condition, is the proximate cause of this disease, M. Herard does not concur, but believes these local injuries effects, not causes.

He then says, "Hence the local derangements can not be taken as a guide, in the choice of medication most appropriate to a fever, in which the collapse of vital power, and obvious tendency to mortification, points most distinctly to a primary alteration in the composition of the blood."

This author then proceeds, in a brief summary, to give the treatment most appropriate in typhoid, in which he assigns the most important place to food: "Despite the wise precepts of Hippocrates, despite the recent researches which have only confirmed their value, we are still all more or less influenced by the now exploded doctrine of irritation. The terms fever, and food, still appear to imply a contradiction, although it is but too certain, that in typhoid, prolonged abstinence leads to the most disastrous results." He then gives the history of a case of typhoid, of alaxic form, accompanied with delirium, vomiting, and diarrhœa, successfully treated with food alone. He found it almost impossible at first to carry out this plan of treatment. It was with the utmost difficulty that a few drops of beef tea were swallowed. He succeeded, however, by dint of perseverance. And when the food remained on the stomach, and in proportion to its increase, the pulse fell from 145 to 130, 120, 115 and 100, the delirium yielded, and, in short, the patient recovered. Again, he says, "Patients who have received adequate support during the progress of typhoid have been known to pass without any transition from disease to health, and walk in the garden of the hospital on the very first day they left their bed."

The abridgement of convalescence in the cases reported by M. Herard seems to illustrate the foregoing pathological views. The diet treatment, by supporting the vital powers, prevents the lesion which otherwise must have occurred by chemical metamorphosis.

Prof. Flint, of New York, says: "In inflammatory affections, proper discrimination is necessary in the employment of the so called antiphlogistic measures, which, if failing to exert a controlling influence, are always hurtful, and it may be, destructive, by impairing the vital forces. Experience also dictates the judicious use of remedies addressed to the internal causative conditions pertaining to the blood; so far as our present knowledge in these most important provinces of pathology and therapeutics will permit. But this is not all, this diathesis often demands that the vital powers be sustained. Sustaining measures of treatment, practically considered, consist of tonics, alcoholic stimulants, and nutritious diet. We will not inquire as to the *rationale* of the operation of these measures; suffice it to say, clinical experience shows abundantly, that they lessen the degree to which the vital forces would otherwise be impaired by disease, and may prevent a fatal termination of disease by asthenia, or exhaustion." Prof. Flint proceeds in an elaborate and very clear style, to show what important changes have been almost universally adopted within a few years past, in the treatment of many diseases, by discarding depletory measures, and adopting instead, stimulants, tonics, and nutritious diet. In reference to bloodletting, he goes on to say, "Clinical observation, which is alone competent to settle the question, has shown that it is a remedy not called for so often, nor to so great an extent in acute inflammations, as was supposed but a few years ago." He makes the following remark in reference to blood-letting, and says it will apply to other measures as well: "Blood-letting may not increase the mortality from a disease, nor protract its continuance, and yet prove injurious. The injury may be manifest only in the slowness of convalescence, and the impaired condition of the system after recovery." And here, perhaps, the same explanation will apply as that given above, to M. Herard's case of convalescence abridged by diet treatment. Again, the same author says, "Every sagacious practitioner knows that certain symptoms, no matter with what disease they are associated, denote failure of the vital powers, or inability to resist disease. He estimates the amount of danger by these symptoms, and often bases his prognostics far more on them than on the nature and extent of the local affection. Every practitioner knows that an inflammation, the same in all respects so far as the local affection is concerned, in different persons, affects the vital forces differently.

Take, for example, pneumonia, extending over the same space, and inducing an equal amount of changes which physical explorations enable us to determine with accuracy. One patient manifests but little disturbance of the system, and no symptoms denoting danger, while another will succumb to the disease. It is also well known, that some persons while in health, show no lack of vigor, yet have very little ability to resist disease. They are quickly destroyed by affections which other persons readily endure, and endure, perhaps, without much inconvenience. Of course, these facts are explicable, but not with our present knowledge; and until explained, it answers to refer them to differences as regards the vital powers, or forces. They are facts of not a little practical importance." Again, he remarks, "Since physicians have ceased to agree with Southwood Smith, in regarding inflammation as an almost concomitant, and the chief source of danger in fever, the importance of preserving and sustaining the powers of life has been more and more appreciated; and at the present moment, with the most intelligent practitioners, these are the leading objects of treatment."

Prof. Flint has been quoted thus at length, because of the apparent coincidence between many of his very just conclusions and the writer's pathological views of malarial diseases. And it is hoped that when the subject of chemico-vital action comes to be better understood, as well as the phenomena of electricity in connection with it, much that is now obscure will have been explained.

The important practical lesson designed to be presented in the foregoing remarks, is to give some clear marks, if possible, by which diseases of malarial origin can be distinguished from diseases arising from other causes. And if this attempt should shed any light on the pathology of these diseases, it must in the same ratio contribute to therapeutics, and, consequently, lead to much greater uniformity in the treatment of a class of diseases, by far the most numerous with which we meet.

It is not clear--as above remarked—that some diseases are not ascribed to malaria, which do not belong to it. And on the other hand, unquestionably, error is often committed by overlooking some clear marks which distinguish malarial from other forms of disease. It is true, few would fail to recognize these marks, in cases like that reported some years ago by Dr. Parry, in a thesis read before the Medical Society in Philadelphia—which is said by some writer, to have fallen like a bombshell at the feet of the professors of the Jefferson Medical College—when he quietly asked, "What shall I do with the pulseless, moribund cases of congestive fever, of the Mississippi?" Possibly, *some* might reply yet, as did Prof. Meigs *then:* "The lancet! the lancet! the lancet!"

It affords much pleasure, however, to believe, that a great majority of physicians *now*, understand these cases better.

Yet, it is believed, by keeping in view the foregoing suggestions, on the effects of malaria, error in diagnosis will much more seldom occur. In the limited experience of the writer

—and, no doubt, in that of most practitioners—cases of fever, and even flux, have been met with where, perhaps, the inattentive observer would have pronounced them malarial; but where, in reality, no such characteristic symptoms were manifest. On the contrary, there was strong, full pulse, furred tongue,—not coated—and no marks denoting depression of the vital powers, and which yielded readily to full antiphlogistic treatment, as blood letting, &c.; attended also, with little, or no convalescence.

On the other hand, cases clearly of inflammatory conditions, as Pneumonia, Pleuritis, or Pleuro-Pneumonia, and other forms of the so-called congestive fevers,—where marks of vital depression were clearly discernable,—have shown such wonderful tolerance of stimulants, that even pints of alcoholic spirits were given daily, with no other apparent effect than to lessen the frequency of the pulse, diminish the heat of the skin, and render the mind more clear; and under this treatment readily recover.

It is apparent then, that diagnostic errors in these cases can only be avoided by careful clinical observation of these morbid symptoms, and not by any attempt at classification of these diseases upon any other basis.

